# Kidney stones and the risk of renal cell carcinoma and upper tract urothelial carcinoma: the Netherlands Cohort Study

**DOI:** 10.1038/s41416-018-0356-7

**Published:** 2018-12-19

**Authors:** Jeroen A. A. van de Pol, Piet A. van den Brandt, Leo J. Schouten

**Affiliations:** 1 Department of Epidemiology, GROW – School for Oncology and Developmental Biology, Maastricht University, Maastricht, The Netherlands; 2 Department of Epidemiology, Care and Public Health Institute (CAPHRI), Maastricht University, Maastricht, The Netherlands

**Keywords:** Cancer epidemiology, Renal cell carcinoma, Risk factors

## Abstract

**Background:**

We examined the association between kidney stones and renal cell carcinoma (RCC) and upper tract urothelial carcinoma (UTUC) risk in the Netherlands Cohort Study on diet and cancer.

**Methods:**

In total, 120,852 participants aged 55–69 completed a self-administered questionnaire on diet, medical conditions and other risk factors for cancer at baseline (1986). After 20.3 years of cancer follow-up 4352 subcohort members, 544 RCC cases and 140 UTUC cases were eligible for case-cohort analysis. Hazard ratios (HR) and 95% confidence intervals (CIs) were estimated by multivariable-adjusted proportional hazards models.

**Results:**

Kidney stones were associated with an increased RCC risk (HR: 1.39, 95% CI 1.05–1.84), vs. no kidney stones. Kidney stones were associated with an increased risk of papillary RCC (HR: 3.08, 95% CI 1.55–6.11), but not clear-cell RCC (HR: 1.14, 95% CI 0.79–1.65). UTUC risk was increased for participants with kidney stones (HR: 1.66, 95% CI 1.03–2.68). No heterogeneity of associations was found for UTUC in the ureter and renal pelvis. An early kidney stone diagnosis (≤40 years) was associated with an increased RCC and UTUC risk, compared to later diagnosis.

**Conclusion:**

Kidney stones were associated with increased papillary RCC risk, but not clear-cell RCC risk. No heterogeneity was found for UTUC subtypes.

## Introduction

Kidney stones, a common urological condition, affect 5–10% of the population in Europe and North America.^1^ Globally, the incidence and prevalence of kidney stones have increased over the years and are expected to increase further through the increasing prevalence of related medical conditions, such as obesity and diabetes mellitus.^1,2^ In general, kidney stone occurrences increase with age and are more common in men than in women.^1,3^ The likelihood of kidney stones decreases with an increased intake of fluids, fruits, and vegetables. Sodium restriction also reduces the probability for kidney stones.^3^

Several studies have assessed the relationship between kidney stones and renal cell carcinoma (RCC) and upper tract urothelial carcinoma (UTUC).^4–6^ Recently, a meta-analysis, based on eight case-control studies and one retrospective cohort study, found an increased risk of RCC and both ureter and renal pelvis cancer in individuals with kidney stones.^7^ Furthermore, kidney stones were associated with an increased risk of RCC in males, but not in females. Three retrospective cohort studies not included in the aforementioned meta-analysis also found an increased risk of renal, ureter, or renal pelvis cancer in patients with urinary tract stones.^8–10^

Increased cancer risks associated with kidney stones are commonly attributed to chronic inflammation and infections, which may lead to an altered proliferation in urothelial cells.^5,11,12^ In turn, this process may lead to the development of a tumour. However, this association may also be explained by shared risk factors between kidney stones and RCC and UTUC.^5,11^ For example, obesity, diabetes mellitus and several dietary factors are also associated with RCC risk.^13,14^

At present, solely case-control and retrospective cohort studies have assessed the relationship between kidney stones and RCC and UTUC risk. These study designs tend to be prone to information and selection bias, which may affect found associations. In addition, most of these studies were limited in their adjustment for confounding factors.^7^ As a result, there is uncertainty whether kidney stones or a lifestyle related to kidney stone formation are associated with an increased RCC and UTUC risk. In this study, we investigate the relationship between self-reported history of kidney stones and the risk of RCC and UTUC in the Netherlands Cohort Study (NLCS) on diet and cancer. In the NLCS, detailed information on risk factors associated with kidney stones, RCC and UTUC has been collected prior to cancer development enabling this study to adjust for multiple confounders.

## Methods

### Study population

The NLCS is a nation-wide prospective cohort study initiated in September 1986. It included 58,279 men and 62,573 women aged 55–69 years at baseline. The study design has been described in detail elsewhere.^15^ In short, the study is a prospective cohort study initiated to investigate the association between diet and the development of cancer. For efficiency in data processing and analysis, a case-cohort design was used. A subcohort of 5000 participants, of which 2411 men and 2589 women, was randomly sampled from the full cohort at baseline to estimate person-time at risk for the entire cohort.^15^

At baseline, all participants completed a mailed, self-administered questionnaire on dietary habits and other risk factors for cancer. By filling in and returning the baseline questionnaire participants agreed to participate in the NLCS. Follow-up for cancer occurrence for all participants was conducted by computerised record linkage with the Netherlands Cancer Registry (NCR), the Netherlands Pathology Registry (PALGA), and causes of death registry maintained by Statistics Netherlands (CBS). In addition, subcohort members were followed-up biannually for migration and vital status by contacting the participants and the municipalities. The completeness of cancer follow-up through record linkage is estimated to be at least 96%.^16^ The institutional review boards of the Netherlands Organization for Applied Scientific Research TNO (Zeist) and Maastricht University (Maastricht) approved the NLCS. The NLCS was conducted in accordance with the Declaration of Helsinki.

In total, 608 RCC cases were identified in the NLCS between 1986 and 2006 (20.3 years). Histologically confirmed epithelial RCC cases were eligible for the collection of formalin-fixed paraffin-embedded (FFPE) tumour tissues. Overall, FFPE tumour tissues were collected for 454 (79.8%) of the eligible cases. Tumour histology was revised by two experienced pathologists according to the WHO-classification of RCC tumours.^17^ Of the 454 RCC cases with available tumour tissue, 366 (80.6%) were clear-cell (cc)RCC cases, 60 (13.2%) papillary (p)RCC cases, 15 (3.3%) chromophobe RCC cases, and 13 (2.9%) other or undefined RCC cases. Further classification of pRCC cases resulted in 35 (7.7%) type 1 pRCC, 24 (5.3%) type 2 pRCC, and 1 (0.2%) undefined pRCC.

**Fig. 1:**
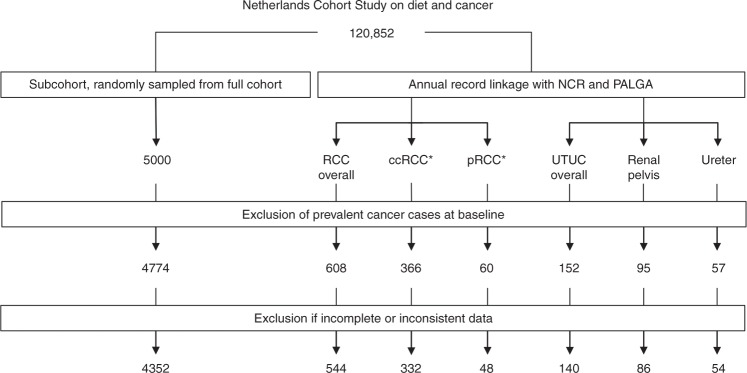
Flow diagram of subcohort members and case subjects on whom the analyses were based. RCC renal cell carcinoma; ccRCC clear-cell renal cell carcinoma; pRCC Papillary renal cell carcinoma; UTUC upper tract urothelial carcinoma; NCR Netherlands Cancer Registry; PALGA the Netherlands Pathology Registry. * Histologically revised subtypes of RCC. Other subtypes were not assessed because of an insufficient number of cases

Cohort members with prevalent cancer at baseline, except skin cancer, and incomplete or inconsistent information on *a priori* selected confounders were excluded from analyses. Figure 1 shows the selection and exclusion of participants. In total, 544 RCC cases (International Classification of Diseases for Oncology 3 (ICD-O-3) C64.9), 140 UTUC cases, and 4352 subcohort members were included in this study. Of eligible RCC cases with confirmed tumour histology 332 were ccRCC cases and 48 were pRCC cases. Of UTUC cases 86 were renal pelvis cancer cases (ICD-O-3 C65.9) and 54 ureter cancer cases (ICD-O-3 C66.9).

### Questionnaire data

All participants completed a mailed, self-administered questionnaire at baseline on dietary habits and other risk factors for cancer. The exposure to kidney stones was obtained from the question “Has a physician ever diagnosed kidney stones and what was your age at that time?”. Participants reported the age at first kidney stone diagnosis in 5-year increments starting from “younger than 30”. Information on dietary habits was obtained through a 150-item, semi-quantitative food frequency questionnaire (FFQ) focusing on habitual consumption of food and beverages during the year preceding baseline. Other risk factors included in the questionnaire considered for the association between RCC, UTUC, and kidney stones were: age at baseline, anthropometry (height and weight), cigarette smoking (status, intensity, and duration), medical conditions (hypertension, diabetes mellitus, and kidney stones including age at first diagnosis in 5-year increments), and the use of diuretic medication.

### Statistical analyses

Cox proportional hazards models were used to estimate age- and sex-adjusted and multivariable-adjusted hazard ratios (HR) and 95% confidence intervals (CIs). *A priori* confounders in the multivariable-adjusted model were body mass index (BMI, kg/m^2^; continuous), hypertension (yes/no), smoking status (never, former, and current), smoking intensity (cig/d, centered; continuous), and smoking duration (years, centered; continuous). Potential confounders were added to the multivariable-adjusted model if they affected the HR of kidney stones on RCC and UTUC risk for more than 10%. Potential confounders considered were diuretic medication use (yes/no), alcohol intake (g/d; continuous), fluid intake (liters; continuous), sodium intake (g/d, adjusted for total energy intake by residuals; continuous), total energy intake (kcal/d; continuous), fruit intake (g/d; continuous), vegetable intake (g/d; continuous), and family history of renal carcinoma (yes/no). None of these potential confounders affected the HR of kidney stones on RCC and UTUC risk by more than 10% and, therefore, none were included in the final multivariable-adjusted models.

Additionally, the relationship between kidney stones and the risk of histological RCC subtypes (ccRCC and pRCC), and UTUC subtypes based on location (renal pelvis cancer and ureter cancer) was analysed. Moreover, the association for age of first reported kidney stone diagnosis was assessed as the main exposure for RCC and UTUC risk. An age of 40 years was determined as cutoff point based on the median age at first kidney stone occurrence.

HRs and 95% CIs were obtained through Cox proportional hazards regression models using Stata statistical software: release 14 (StataCorp., 2015, College Station, TX). Person-years at risk were calculated from baseline until registration of RCC or UTUC (depending on the cancer of interest) or until date of censoring by death, emigration, loss to follow-up or end of follow-up, whichever occurred first. During analyses for RCC or UTUC solely the cancer of interest was considered as an event and the development of other cancer types did not lead to censoring of observations. During analyses with histological RCC subtypes or UTUC subtypes based on location as the outcome, the first occurring subtype was considered as the time of censoring. The proportional hazards assumption was tested with Scaled Schoenfeld residuals and log–log curves.^18^ Age at baseline was included as a time-varying covariate due to a violation of this assumption. Additional variance introduced due to the case-cohort approach by sampling a subcohort from the cohort was accounted for by use of the Huber-White sandwich estimator for standard errors, similar to the variance-covariance estimator by Barlow.^19^

Tests for heterogeneity were performed to evaluate differences between histological subtypes and different localizations of tumours using the competing risks procedure in Stata. *P*-values were calculated based on a bootstrapping method that was developed for the case-cohort design. This procedure has been described in detail elsewhere.^20,21^

All tests were performed two-sided and *P*-values <0.05 were considered statistically significant.

### Results

Subcohort baseline characteristics categorised by history of kidney stones are presented in Table 1. In total, 8.4% of the subcohort had a history of kidney stones. In general, baseline characteristics for members with a history of kidney stones were similar to members without a history of kidney stones. Exceptions included a decreased proportion of current smokers in males with kidney stones and an increased fruit and vegetable intake in both men and women with kidney stones, when compared to participants without kidney stones in those categories.

**Table 1:** Characteristics of subcohort members categorized by history of kidney stones; the Netherlands Cohort Study on diet and cancer, 1986–2006

Baseline characteristics^a^	Men		Women		Total	
	History of kidney stones		History of kidney stones		History of kidney stones	
	Yes	No	Yes	No	Yes	No
Number of persons, *n* %	267 (12.9)	1811 (87.2)	98 (4.3)	2176 (95.7)	365 (8.4)	3987 (91.6)
*Exposures*						
Age at first kidney stone, y	42.2 (11.3)	—	43.0 (13.5)	—	43.0 (10.9)	—
Time since first occurrence of kidney stones, y^b^	19.2 (11.5)	—	19.8 (12.9)	—	18.8 (10.9)	—
*Covariates*						
Age at baseline, y	60.9 (4.0)	61.3 (4.2)	62.3 (4.3)	61.5 (4.3)	61.3 (4.1)	61.4 (4.2)
BMI, kg/m^2^	25.2 (2.8)	25.0 (2.6)	24.9 (3.3)	25.1 (3.6)	25.1 (2.9)	25.1 (3.1)
*Cigarette smoking status, n %*						
Never	39 (14.6)	250 (13.8)	58 (59.2)	1312 (60.3)	97 (26.6)	1562 (39.2)
Former	150 (56.2)	914 (50.5)	17 (17.4)	428 (19.7)	167 (45.8)	1342 (33.7)
Current	78 (29.2)	647 (35.7)	23 (23.5)	436 (20.0)	101 (27.7)	1083 (27.2)
*Ever cigarette smokers only*						
Smoking duration, y	32.8 (11.6)	33.8 (11.7)	27.2 (14.0)	27.9 (12.3)	32.0 (12.1)	31.7 (12.2)
Smoking intensity, cig/d	17.4 (11.1)	17.1 (10.5)	13.0 (8.9)	11.7 (8.3)	16.7 (10.9)	15.2 (10.1)
Family history of renal cancer, *n* %	2 (0.8)	12 (0.7)	1 (1.0)	31 (1.4)	3 (0.8)	43 (1.1)
Hypertension, *n* %	67 (25.1)	425 (23.5)	31 (31.6)	641 (29.5)	98 (26.9)	1066 (26.7)
Diabetes mellitus, *n* %	7 (2.6)	64 (3.5)	6 (6.1)	86 (4.0)	13 (3.6)	150 (3.8)
Use of diuretic medication, *n* %	29 (10.9)	140 (7.7)	15 (15.3)	309 (14.2)	44 (12.1)	449 (11.3)
Fluid intake, L/d	1.5 (0.5)	1.5 (0.5)	1.3 (0.4)	1.3 (0.4)	1.4 (0.5)	1.4 (0.4)
Alcohol intake, g/d^c^	17.1 (16.1)	17.6 (17.2)	8.1 (10.3)	8.7 (10.4)	15.0 (15.4)	13.4 (15.1)
Sodium intake, g/d	2.6 (0.9)	2.6 (0.9)	2.1 (0.7)	2.1 (0.7)	2.5 (0.9)	2.3 (0.8)
Fruit intake, g/d	157.3 (118.6)	153.7 (115.4)	196.5 (121.3)	194.6 (123.2)	167.8 (120.4)	176.1 (121.4)
Vegetable intake, g/d	194.1 (78.5)	190.9 (87)	202.0 (94.7)	192.8 (82.9)	196.2 (83.1)	191.9 (84.8)

^a^Data represent mean values (SD); for categorical variables *N* (%) is presented. Numbers may not add up to 100% as a result of missing values. Solely participants with complete information on the main exposures and the *a priori* selected confounders are presented in this table

^b^Time between reported age of first kidney stone occurrence and age at baseline

^c^In consumers only

Results from age- and sex-adjusted analyses did not differ substantially from the multivariable-adjusted results. Tables 2 and 3 present the results of the multivariable-adjusted analyses on history of kidney stones and age at first diagnosis of kidney stones for RCC and UTUC, respectively. In multivariable-adjusted analyses, participants with a history of kidney stones had a statistically significantly increased overall RCC risk (Table 2, HR: 1.39, 95% CI 1.05–1.84) compared to participants without a history of kidney stones. Kidney stones were significantly associated to pRCC (HR: 3.08, 95% CI 1.55–6.11), whereas no association was found for ccRCC (HR: 1.14, 95% CI 0.79–1.65). Tests for heterogeneity of associations for kidney stones indicated significant differences between ccRCC and pRCC for all participants (*P* = 0.001). Type 1 pRCC risk and type 2 pRCC risk did not differ substantially from overall pRCC estimates (data not shown). In males, similar findings were obtained for RCC overall (HR: 1.42, 95% CI 1.04–1.93), pRCC (HR: 2.37, 95% CI 1.16–4.85) and ccRCC (HR: 1.20, 95% CI 0.80–1.81). In females, no statistically significant association was found for RCC overall (HR: 1.30, 95% CI 0.68–2.50) and ccRCC (HR: 0.88, 95% CI 0.35–2.22). An increased risk of pRCC (HR: 16.37, 95% CI 3.53–75.89) was found for females with a history of kidney stones compared to females without kidney stones. However, in females, the number of exposed cases was limited.

**Table 2:** Associations between kidney stones and risk of renal cell carcinoma subtypes; Netherlands Cohort Study on diet and cancer, 1986–2006

	Subcohort person-time at risk (y)	RCC overall^a^		ccRCC^a^		pRCC^a^		
		No. of cases	HR (95% CI)	No. of cases	HR (95% CI)	No. of cases	HR (95% CI)	*P*. heterogeneity^f^
Unadjusted analyses								
History of kidney stones								
*All participants* ^b^								
No	67,294	473	1 (reference)	296	1 (reference)	34	1 (reference)	
Yes	5982	71	1.40 (1.06–1.85)	36	1.15 (0.80–1.66)	14	3.20 (1.64–6.25)	0.001
Multivariable-adjusted analyses								
History of kidney stones								
*All participants* ^c^								
No	67,294	473	1 (reference)	296	1 (reference)	34	1 (reference)	
Yes	5982	71	1.39 (1.05–1.84)	36	1.14 (0.79–1.65)	14	3.08 (1.55–6.11)	0.001
*P* for interaction^d^			0.815		0.593		0.022	
*Males only* ^e^								
No	28,706	285	1 (reference)	172	1 (reference)	30	1 (reference)	
Yes	4285	60	1.42 (1.04–1.93)	31	1.20 (0.80–1.81)	11	2.37 (1.16–4.85)	0.084
*Females only* ^e^								
No	38,588	188	1 (reference)	124	1 (reference)	4	1 (reference)	
Yes	1697	11	1.30 (0.68–2.50)	5	0.88 (0.35–2.22)	3	16.37 (3.53–75.89)	0.756
Age at first occurrence of kidney stones (participants with kidney stones only)								
*All participants* ^c^								
<40	2354	40	2.10 (1.21–3.65)	17	1.43 (0.70–2.93)	9	3.52 (0.95–13.01)	0.854
≥40	3553	31	1 (reference)	19	1 (reference)	5	1 (reference)	

^a^*RCC* renal cell carcinoma, *Overall* no selection on morphological subtypes, *ccRCC* clear-cell renal cell carcinoma, *pRCC* papillary renal cell carcinoma

^b^Model adjusted for age at baseline (y), sex (male/female), and age at baseline (y) as a time-varying covariate

^c^Model adjusted for age at baseline (y), sex (male/female), body mass index (kg/m^2^), smoking status (never, former, current), smoking intensity (cig/d, centered), smoking duration (y, centered), hypertension (yes, no), and age at baseline (y) as a time-varying covariate

^d^Test on whether sex modifies the (multivariable-adjusted) relationship between the history of kidney stones and RCC risk, based on the Wald test for the interaction term

^e^Model adjusted for age at baseline (y), body mass index (kg/m^2^), smoking status (never, former, current), smoking intensity (cig/d, centered), smoking duration (y, centered), hypertension (yes, no), and age at baseline (y) as a time-varying covariate

^f^Test for heterogeneity between histological RCC subtypes

**Table 3:** Associations between kidney stones and risk of upper tract urothelial carcinoma localizations; Netherlands Cohort Study on diet and cancer, 1986–2006

	Subcohort person-time at risk (y)	UTUC overall^a^		Renal pelvis		Ureter		
		No. of cases	HR (95% CI)	No. of cases	HR (95% CI)	No. of cases	HR (95% CI)	*P*. heterogeneity^f^
Unadjusted analyses								
History of kidney stones								
*All participants* ^b^								
No	67,389	118	1 (reference)	72	1 (reference)	46	1 (reference)	
Yes	5981	22	1.64 (1.02–2.64)	14	1.74 (0.95–3.16)	8	1.49 (0.70–3.16)	0.841
Multivariable-adjusted analyses								
History of kidney stones								
*All participants* ^c^								
No	67,389	118	1 (reference)	72	1 (reference)	46	1 (reference)	
Yes	5981	22	1.66 (1.03–2.68)	14	1.76 (0.96–3.23)	8	1.50 (0.71–3.18)	0.841
*P* for interaction^d^			0.923		0.395		—^g^	
*Males only* ^e^								
No	28,774	81	1 (reference)	49	1 (reference)	32	1 (reference)	
Yes	4284	19	1.65 (0.98–2.79)	11	1.59 (0.81–3.14)	8	1.74 (0.80–3.80)	0.835
*Females only* ^e^								
No	38,615	37	1 (reference)	23	1 (reference)	14	1 (reference)	
Yes	1697	3	1.70 (0.51–5.72)	3	2.77 (0.80–9.57)	0	—^g^	—
Age at first occurrence of kidney stones (participants with kidney stones only)								
*All participants* ^c^								
<40	2354	11	1.76 (0.69–4.52)	5	1.13 (0.30–4.22)	6	4.79 (0.83–27.71)	0.892
≥40	3552	11	1 (reference)	9	1 (reference)	2	1 (reference)	

^a^*UTUC* Upper Tract Urothelial Carcinoma, *Overall* no selection on subtypes by localization

^b^Model adjusted for age at baseline (y), sex (male/female), and age at baseline (y) as a time-varying covariate

^c^Model adjusted for age at baseline (y), sex (male/female), body mass index (kg/m^2^), smoking status (never, former, current), smoking intensity (cig/d, centered), smoking duration (y, centered), hypertension (yes, no), and age at baseline (y) as a time-varying covariate

^d^Test on whether sex modifies the (multivariable-adjusted) relationship between the history of kidney stones and UTUC risk, based on the Wald test for the interaction term

^e^Model adjusted for age at baseline (y), body mass index (kg/m^2^), smoking status (never, former, current), smoking intensity (cig/d, centered), smoking duration (y, centered), hypertension (yes, no), and age at baseline (y) as a time-varying covariate

^f^Test for heterogeneity between renal pelvis and ureter subtypes

^g^Insufficient number of exposed cases

A history of kidney stones was statistically significantly associated with an increased risk of UTUC overall in multivariable-adjusted models (Table 3, HR: 1.66, 95% CI 1.03–2.68). Similar estimates were found for specific UTUC localizations, namely the renal pelvis (HR: 1.76, 95% CI 0.96–3.23) and the ureter (HR: 1.50, 95% CI 0.71–3.18). Tests for heterogeneity of associations did not indicate significant differences between cancer of the renal pelvis and the ureter (*P* = 0.841). Associations were similar for sex-specific estimates for UTUC overall and UTUC localizations. Due to the absence of exposed cases, no analyses on ureter cancer were performed in females.

An early diagnosis of kidney stones (<40 years), compared to a later kidney stone diagnosis (≥40 years), was statistically significantly associated with an increased overall RCC risk (HR: 2.10, 95% CI 1.21–3.65). For histological RCC subtypes a non-statistically significantly increased risk was found for both pRCC (HR: 3.52, 95% CI 0.95–13.01) and ccRCC (HR: 1.43, 95% CI 0.70–2.93). In UTUC analyses, an association was found for age (<40 years) at first diagnosis of kidney stones and overall UTUC risk (HR: 1.76, 95% CI 0.69–4.52). Whereas no association was found for UTUC localized in the renal pelvis (HR: 1.13, 95% CI 0.30–4.22), an increased risk of ureter cancer was found (HR: 4.79, 95% CI 0.83–27.71). However, the number of participants was limited for this analysis, which reduced the power to find statistically significant results.

## Discussion

In this study, an increased RCC and UTUC risk was found for participants with a history of kidney stones. Moreover, an increased pRCC risk, but not ccRCC risk, was observed in relation to a history of kidney stones. Furthermore, an increased RCC and UTUC risk was found for participants with a kidney stone diagnosis before their fortieth birthday. To our knowledge, this is the first prospective study to examine the relationship between kidney stones and RCC and UTUC risk and the first study to show heterogeneity of associations between pRCC and ccRCC.

The present study concurs with previously published studies on the relationship between kidney stones and RCC and UTUC risk. In a meta-analysis by Cheungpasitporn et al. an overall risk ratio of 1.76 (95% CI 1.24–2.49) was found comparing RCC risk for patients with kidney stones to those without kidney stones.^7^ In the same meta-analysis a pooled risk ratio of 2.14 (95% CI 1.35–3.40) was found for transitional cell carcinoma, involving the ureter and renal pelvis.^7^ Although HR estimates are lower in our study, we found similar associations for both RCC and UTUC risk. In addition, Cheungpasitporn et al. found an increased RCC risk associated with kidney stones in males, but not females.^7^ In contrast, we found no difference between males and females.

To our knowledge, this is the first study to find an increased pRCC risk in participants with kidney stones. Nearly half of all pRCC cases in our study could be attributed to kidney stones based on the population attributable fraction using a multivariable-adjusted HR of 3.08.^22^ In general, pRCC is a heterogeneous RCC subtype consisting of two distinct subtypes characterised by genetic variations in the *MET*-gene for type 1 pRCC and in fumarate hydratase for type 2 pRCC.^17,23^ Previous studies on kidney stones have often been unable to assess the relationship with pRCC, either because pRCC has only been classified as a distinct tumour type since 1996, or because they did not contain information on tumour histology.^24^ Our study was able to assess tumour histology through centralised revision by two pathologists. Even though pRCC is a very heterogeneous subtype of RCC, we did not find differences between type 1 pRCC and type 2 pRCC.

There is uncertainty regarding the biological mechanism that may relate kidney stones to kidney cancer. Kidney stones are presumed to cause chronic irritation in the local environment of the kidney and ureter.^5–7^ In general, chronic irritation and infection recruit inflammatory cells, which secrete cytokines and chemokines. In turn, free radical species from oxygen and nitrogen are produced, facilitating the onset of cancer through, among others, increased cell proliferation.^12^ However, more insight on the role of kidney stones in this process is needed to elucidate the found associations.

Chow et al. found that most renal pelvis and ureter cancers occurred on the same side as kidney stone formation, which could indicate that kidney stones are exerting the effect in UTUC.^5^ In animal studies, induced stone formation was correlated to the development of bladder cancer.^25,26^ By suppressing stone formation in rats, the effect of kidney stones could be attributed to the irritative stimulation by kidney stones, rather than to metabolites of the stone inducing factor.^25^ However, both ccRCC and pRCC are thought to originate from the proximal convoluted renal tubule.^23^ It is deemed unlikely that stones or stone-forming crystals deposit in the proximal convoluted renal tubule. Kidney stones tend to form in locations where there is a combination of supersaturation of the urine and where there is a change in the luminal diameter of the renal tubules, such as the loop of Henle, the distal tubules and in the collecting ducts.^27^ Therefore, urinary solutes or a predisposing lifestyle, rather than actual stone formation in the kidney, might play a role in the development of these cancer subtypes. In contrast to pRCC, ccRCC risk was not associated with a history of kidney stones in this study. In general, genetic susceptibility and the interaction with environmental exposures are believed to influence RCC risk.^28^ Hypothetically, tumour development could be related to the presence of stone-forming salts in the filtrate of the proximal tubules. The presence of these solutes may affect cell metabolism, which could potentially result in the development of distinct renal cancer subtypes.

UTUC risk was increased in participants with kidney stones, compared to participants without kidney stones, but no difference was found between the localization in the renal pelvis or the ureter. In contrast to the proximal tubule, stone formation is common in the renal pelvis and ureter, which enables kidney stones to cause chronic irritation and inflammation to urothelial cells. In turn, this may explain the increased UTUC risk in relation to kidney stones.

In this study, an age below 40 years at first kidney stone diagnosis was potentially associated with an increased RCC and UTUC risk. However, further research on this potential association is needed as the number of cases eligible for these analyses was limited. An earlier kidney stones diagnosis could provide a longer time period for kidney stones to induce chronic irritation to the local environment or for potentially harmful solutes in the urine to have a carcinogenic effect. Therefore, the found associations could indicate that the lifestyle of kidney stone formers may already play a role in the development of cancer during the early stages of life.

The strengths of the present study were the complete follow-up, the extensive information on potential confounders and the differentiation between histological subtypes based on the centralised revision by experienced pathologists.

However, this study was also subject to limitations. Information on kidney stones was retrieved from a self-administered questionnaire at baseline. Consequentially, kidney stone occurrences beyond the age at baseline may have been missed for participants with and without cancer. However, peak kidney stone incidence is expected at 40–49 years of age. Therefore, effects on our results are assumed to be limited. Furthermore, information obtained through self-reported questionnaires may contain inaccuracies regarding the diagnosis of kidney stones. However, the prevalence and incidence of kidney stones were as expected in the population and the RCC and UTUC risk was the greatest before 40 years of age.^1^ Therefore, we think that our results are generalizable for the population. In this study, we did not have information on kidney stone composition, frequency and laterality. This information could provide additional insight on the mechanisms behind the found association in future studies. Residual confounding could have affected our results. However, as all models were extensively adjusted for confounders we expect this effect to be limited. Finally, a diagnosis of kidney stones may warrant additional surveillance, which could lead to an earlier detection of RCC. However, in our study, the average tumour size was larger in cases with a history of kidney stones, compared to cases without a history of kidney stones (72 mm vs. 65 mm, respectively), which makes bias due to earlier detection unlikely.

In light of the findings of this study, more research is needed to unravel the mechanisms behind the relation of kidney stones and RCC and UTUC. First, future studies are required to ascertain the relationship between kidney stones and pRCC. Second, more studies are needed on kidney stone composition, stone laterality and exposure to stone-forming solutes to uncover the impact on cell metabolism and cancer development. Finally, more studies are required to get a better insight on sex-specific differences in RCC and UTUC risk as a result of kidney stones.

## References

[1] Romero V, Akpinar H, Assimos DG (2010). Kidney stones: a global picture of prevalence, incidence, and associated risk factors. Rev. Urol..

[2] Sfoungaristos S, Gofrit ON, Yutkin V, Pode D, Duvdevani M (2015). Prevention of renal stone disease recurrence. A systematic review of contemporary pharmaceutical options. Expert. Opin. Pharmacother..

[3] Moe OW (2006). Kidney stones: pathophysiology and medical management. Lancet.

[4] McCredie M, Stewart JH (1992). Risk factors for kidney cancer in New South Wales, Australia. II. Urologic disease, hypertension, obesity, and hormonal factors. Cancer Causes Control.

[5] Chow WH (1997). Risk of urinary tract cancers following kidney or ureter stones. J. Natl Cancer Inst..

[6] Chung SD, Liu SP, Lin HC (2013). A population-based study on the association between urinary calculi and kidney cancer. Can. Urol. Assoc. J..

[7] Cheungpasitporn W (2015). The risk of kidney cancer in patients with kidney stones: a systematic review and meta-analysis. QJM.

[8] Sun LM (2013). Urinary tract stone raises subsequent risk for urinary tract cancer: a population-based cohort study. BJU Int..

[9] Shih CJ (2014). Urinary calculi and risk of cancer: a nationwide population-based study. Medicine.

[10] Lin CL (2016). Associations between interventions for urolithiasis and urinary tract cancer among patients in Taiwan: the effect of early intervention. Medicine.

[11] Schlehofer B (1996). International renal-cell-cancer study. VI. the role of medical and family history. Int. J. Cancer.

[12] Federico A, Morgillo F, Tuccillo C, Ciardiello F, Loguercio C (2007). Chronic inflammation and oxidative stress in human carcinogenesis. Int. J. Cancer.

[13] Stewart JH (2003). Cancers of the kidney and urinary tract in patients on dialysis for end-stage renal disease: analysis of data from the United States, Europe, and Australia and New Zealand. J. Am. Soc. Nephrol..

[14] McLaughlin, J. K., Lipworth, L., Tarone, R. E., Blot, W. J. Renal Cancer. In *Cancer Epidemiology and Prevention* 3rd edn, (eds Schottenfeld, D., Fraumeni, J. F.) 1087–1100 (Oxford University Press, New York, 2006).

[15] van den Brandt PA (1990). A large-scale prospective cohort study on diet and cancer in The Netherlands. J. Clin. Epidemiol..

[16] Goldbohm RA, van den Brandt PA, Dorant E (1994). Estimation of the coverage of dutch minicipalities by cancer registries and PALGA based on hospital discharge data. Tijdschr. Soc. Gezondh..

[17] Eble JN, Sauter G, Epstein JI, Sesterhenn IA (2004). World Health Organization classification of Tumours. Pathology and Genetics of Tumours of the Urinary System and Male Genital Organs..

[18] Schoenfeld D (1982). Partial residuals for the proportional hazards regression model. Biometrika.

[19] Barlow WE (1994). Robust variance estimation for the case-cohort design. Biometrics.

[20] de Vogel S (2008). Associations of dietary methyl donor intake with MLH1 promoter hypermethylation and related molecular phenotypes in sporadic colorectal cancer. Carcinogenesis.

[21] Wacholder S, Gail MH, Pee D, Brookmeyer R (1989). Alternative variance and efficiency calculations for the case-cohort design. Biometrika.

[22] Rockhill B, Newman B, Weinberg C (1998). Use and misuse of population attributable fractions. Am. J. Public Health.

[23] Shuch B (2015). Understanding pathologic variants of renal cell carcinoma: distilling therapeutic opportunities from biologic complexity. Eur. Urol..

[24] Delahunt B (2001). Morphologic typing of papillary renal cell carcinoma: comparison of growth kinetics and patient survival in 66 cases. Hum. Pathol..

[25] Ogasawara H (1995). Urinary bladder carcinogenesis induced by melamine in F344 male rats: correlation between carcinogenicity and urolith formation. Carcinogenesis.

[26] Okumura M (1992). Relationship between calculus formation and carcinogenesis in the urinary bladder of rats administered the non-genotoxic agents thymine or melamine. Carcinogenesis.

[27] Khan SR (2006). Renal tubular damage/dysfunction: key to the formation of kidney stones. Urol. Res..

[28] Chow WH, Dong LM, Devesa SS (2010). Epidemiology and risk factors for kidney cancer. Nat. Rev. Urol..

